# Andexanet alfa for oral fxa inhibitor-associated major acute intracerebral hemorrhage: insights into clinically relevant thromboembolic events from the ANNEXA-I study

**DOI:** 10.1590/1677-5449.202401332

**Published:** 2025-01-20

**Authors:** Mateo Porres-Aguilar, Luis Antonio Meillon-Garcia, João Carlos de Campos Guerra

**Affiliations:** 1 Texas Tech University Health Sciences Center, Department of Internal Medicine, Divisions of Hospital and Adult Thrombosis Medicine, El Paso, Texas, USA.; 2 Centro Médico ABC, México City, México.; 3 Hospital Israelita Albert Einstein, Laboratório de Hemostasia e Coagulação, São Paulo, SP, Brasil.

Over the past 15 years, there has been a significant shift from vitamin K antagonists (VKAs) to direct oral anticoagulants (DOACs), including the oral factor Xa (FXa) inhibitors apixaban, rivaroxaban, and edoxaban, which are being increasingly used in clinical practice for the prevention and management of venous thromboembolism (VTE) and cardioembolic stroke prevention in atrial fibrillation (SPAF).^1,2^ Although DOACs have been associated with lower risk of fatal and intracranial hemorrhage (ICH) compared to VKAs, the risk of serious bleeding still remains an important side effect.^3^

Rapid reversal with specific agents like Andexanet alfa (AA) may be clinically indicated for acute major severe, life-threatening bleeding events induced by DOACs, or if patients require emergent surgery or invasive surgical procedures. AA is a specific oral FXa inhibitor reversal agent and is a recombinant FXa variant without enzymatic activity, which rapidly decreases anti FXa activity and free drug levels, increasing thrombin generation.^4^ Prothrombin complex concentrate (PCC) is a non-specific reversal agent for DOACs and VKAs and is recognized in the therapeutic armamentarium for DOAC-associated major bleeding events, if specific reversal agents like AA are not available.^4^ 4-factor (4F) PCC contains all four of the vitamin K dependent coagulation proteins (factors II, VII, IX, and X) with small amounts of protein C and S and is the agent most studied for management of DOAC-associated bleeding events.^4^

4F-PCC may increase the risk of thrombosis, since 4F-PCC can increase the level of factor IX, X, and prothrombin with increased production of FXa and, mainly, FIIa, thus increasing thrombin generation.^5^ Most importantly, AA influences the coagulation cascade beyond the inactivation of FXa inhibitors. AA binds and inhibits endogenous FXa inhibitors, more specifically to tissue factor pathway inhibitor (TFPI), activating factor VII/tissue factor, and AA also binds/inhibits the protease inhibitor antithrombin. These additional effects of AA increase the risk for significant thromboembolic (TE) events.^5,6^

The ANNEXA-4 was a prospective, open-label, single-cohort study that examined the effects of AA for treatment of major acute bleeding events in patients receiving FXa inhibitors within 18 hours in 479 patients. 81% of patients were anticoagulated for cardioembolic SPAF; TE occurred in 50 (10%) patients; in 16 of them, these occurred while receiving parenteral thromboprophylaxis; no TE occurred after oral anticoagulation was restarted. It is important to mention that the absence of a control arm limits the interpretation of such safety outcomes.^7^

The ANNEXA-I was a multicenter, randomized 1:1 ratio, prospective study in 530 patients who had taken FXa inhibitors within 15 hours before having an acute ICH allocated to receive either AA or usual care, including use of 4F-PCC. The primary endpoint was hemostatic efficacy, defined as expansion of the intracranial hematoma volume by 35% or less at 12 hours after baseline, an increase in the score on the NIH Stroke Scale < 7 points at 12 hours, and no receipt of rescue therapy between 3 hours and 12 hours (e.g. evacuation craniotomy or endovascular percutaneous intervention). Safety end points were TE events and death. In 267 patient receiving usual care, 85% received PCC. Hemostatic efficacy was achieved in 150/224 patients (67.0%) receiving AA and in 121/228 (53.1%) receiving usual care (95% confidence interval [CI], 4.6 to 22.2; P = 0.003). Interestingly, TE occurred in 27/263 patients (10.3%) receiving AA and in 15/267 (5.6%) receiving usual care (95% CI, 0.1 to 9.2; P = 0.048). It is important to emphasize that TE events, mainly driven by arterial thromboembolism like transient ischemic attack (TIA) and ischemic stroke, occurred in 17 patients (6.5%) versus 4 patients (1.5%), respectively; and myocardial infarction occurred in 11 patients (4.2%) versus 4 patients (1.5%), respectively. Investigators concluded that AA in patients with FXa-inhibitor associated ICH resulted in better control of hematoma expansion than usual care but was associated with increased TE events, mainly driven by TIA/ischemic stroke and myocardial infarction.^8^

We would like to share the following concerns/questions for the investigators who participated in the ANNEXA-I study: Were the investigators able to identify *“high baseline risk”* patients in whom the risk was unacceptably high for thromboembolic events (e.g. CHA_2_DS_2_-VASc Score > 7 points for cardioembolic SPAF)? Is there any correlation between delaying the time when FXa inhibitors are reinitiated and the development of TE events? Could the authors describe in greater detail potential *“red flag signals”* or clinical predictors for those patients at the highest risk for thromboembolic events? Thus, to provide aggressive anticoagulant and/or antiplatelet thromboprophylaxis strategies in a timely and patient-centered individualized manner. We believe there is a lack of data and uncertainty about whether, how, and when to restart oral FXa inhibitors after acute major bleeding events, particularly when neutralization is used; perhaps, these are need gaps that merit further prospective research.

There are still many existing requirements for future directions in this fascinating field: well-designed prospective studies are needed and eagerly awaited to establish the efficacy and safety of 4F-PCC in FXa inhibitor-associated major bleeding events. AA must be rigorously clinically evaluated in head-to-head studies with 4F-PPC, such as in patients taking FXa inhibitors and needing urgent surgery or invasive procedures, for instance. Other important areas to explore include thrombin generation assays and viscoelastic testing as surrogate biomarkers of efficacy and safety and to provide objective knowledge of the baseline and post-treatment pro-thromboembolic or hemostatic status of such patients. However, currently such tests are not yet routinely available in our clinical practice. Point of care assays to measure DOAC concentrations are currently under evaluation and in clinical development.^9^

Finally, it remains to be proven in large-scale, multinational, multicenter, phase 3, randomized clinical trials, whether diverse classes of the newer FXI/XIa inhibitors will be safer, when compared to either enoxaparin and/or oral FXa inhibitors.^10,11^ Moreover, because no patient is exempt from undesirable side effects like bleeding events with such novel anticoagulants, including the newer FXI/XIa inhibitors; thus, reversal strategies for dealing with such situations merit further research and there is a long way to go.^4,12^
